# Prevalence of dysautonomia in chronic musculoskeletal pain: a cross-sectional study

**DOI:** 10.1093/rheumatology/keag511

**Published:** 2026-09-16

**Authors:** Norah A Almutairi, Darren C Greenwood, Manoj Sivan

**Affiliations:** Academic Department of Rehabilitation Medicine, Leeds Institute of Rheumatic and Musculoskeletal Medicine, University of Leeds, Leeds, UK; Physical Therapy and Rehabilitation Department, College of Applied Medical Sciences, Majmaah University, Majmaah, Saudi Arabia; Leeds Institute for Cardiovascular and Metabolic Medicine, School of Medicine, University of Leeds, Leeds, UK; Leeds Institute for Data Analytics, University of Leeds, Leeds, UK; Academic Department of Rehabilitation Medicine, Leeds Institute of Rheumatic and Musculoskeletal Medicine, University of Leeds, Leeds, UK; National Demonstration Centre of Rehabilitation Medicine, Leeds Teaching Hospitals NHS Trust, Leeds, UK

**Keywords:** autonomic dysfunction, dysautonomia, chronic musculoskeletal pain, FM, RA, OA

## Abstract

**Objectives:**

There is substantial evidence that the autonomic nervous system plays a crucial role in pain modulation and chronicity, but the actual prevalence of dysautonomia in musculoskeletal conditions remains unknown. This study investigated the prevalence of dysautonomia in patients with chronic musculoskeletal pain (CMP) compared with healthy controls, quantified the dysautonomia burden in inflammatory and non-inflammatory musculoskeletal conditions, and evaluated the associations between pain severity, psychological factors and dysautonomia severity.

**Methods:**

This cross-sectional study recruited patients with CMP between October 2024 and July 2025 along with healthy participants with similar age-sex distribution. Inclusion criteria were a clinician-confirmed musculoskeletal condition and pain duration >12 weeks. Assessments included the Composite Autonomic Symptom Score-31, the Brief Pain Inventory and the Hospital Anxiety and Depression Scale. A COMPASS-31 score of 17 or higher was considered indicative of dysautonomia.

**Results:**

Of 896 patients screened, 342 participated, along with 57 healthy controls. Dysautonomia was significantly more prevalent in CMP patients (74%) compared with controls (11%) (adjusted RR = 7.4, 95% CI 1.8–13.1, *P* = 0.010). Dysautonomia rates were similarly high in inflammatory (76%) and non-inflammatory (72%) conditions. Risk of dysautonomia increased with pain severity (RR 1.27 per point on BPI, 95% CI 1.08–1.45, *P* < 0.001), depression (RR 1.12 per HADS depression score, 95% CI 1.06–1.19, *P* < 0.001) and anxiety (RR 1.15 per HADS anxiety score, 95% CI 1.08–1.22, *P* < 0.001), adjusting for age and gender.

**Conclusion:**

Dysautonomia is highly prevalent among patients with CMP irrespective of inflammatory status and is associated with pain severity, depression and anxiety. CMP patients should be assessed for dysautonomia as a potential contributor to symptom burden.

*Rheumatology* key messages

- Dysautonomia was over seven times as likely in chronic musculoskeletal pain compared to healthy controls.
- Nearly three-quarters of chronic musculoskeletal pain patients reported dysautonomia symptoms, including inflammatory and non-inflammatory conditions.
- Routine dysautonomia screening should be considered in patients with chronic musculoskeletal pain.

## Introduction

Chronic musculoskeletal pain (CMP) is a major public health problem that affects 1.75 billion people worldwide [1]. This condition is characterized by pain in bones, joints, muscles or related soft tissue that lasts for more than 3 months [2]. It represents a major burden on individuals and health care systems, leading to high healthcare costs and disability rates globally [3, 4]. Recently, the International Association for the Study of Pain (IASP) has recommended treating chronic pain as a primary disease rather than a secondary symptom, in recognition of its significant impact [5].

Chronic pain has complex pathophysiology, with multiple body systems contributing to it. The autonomic nervous system (ANS) plays a significant role in processing and perceiving pain through its sympathetic and parasympathetic branches. It regulates blood flow and tissue perfusion, inflammatory mediator release, descending pain modulation pathways, tissue repair processes and the hypothalamic–pituitary–adrenal axis [6, 7]. In cases of autonomic dysfunction (dysautonomia), these complex regulatory processes can be disrupted, leading to persistent inflammation and pain chronicity [8].

Dysautonomia can affect multiple organ systems. It is characterized by orthostatic intolerance, dizziness, palpitations, gastrointestinal disturbance, bladder symptoms, abnormal sweating and pupillary and vasomotor change [9, 10]. These symptoms overlap considerably with those reported in CMP and have been increasingly recognized across musculoskeletal conditions particularly FM, where they are associated with greater symptom severity [11, 12]. However, the relationship between dysautonomia and the broader CMP population, spanning both inflammatory and non-inflammatory conditions, remains poorly characterized. We therefore aimed to estimate the prevalence and the burden of dysautonomia, among individuals with CMP using COMPASS-31, a validated self-reported questionnaire that assesses six autonomic domains.

## Methods

### Participants

Individuals with CMP attending rheumatology, rehabilitation or orthopaedic clinics were identified during their routine clinic visits and invited to take part in the study. Recruitment took place at Chapel Allerton Hospital (Leeds, UK) between October 2024 and July 2025. The inclusion criteria were: (1) a clinical diagnosis of a musculoskeletal condition with pain lasting at least 12 weeks and not explained by another medical condition and (2) the ability to read and understand English. Exclusion criteria were: (1) inability to provide informed consent, (2) advanced heart disease (including severe heart failure, recent myocardial infarction or current investigations for arrhythmias) and (3) pregnancy. In addition, 57 healthy controls with a similar age–sex distribution were recruited from hospital and university staff, and from patients’ companions. Controls were screened to confirm the absence of CMP.

### Musculoskeletal conditions

Both inflammatory and non-inflammatory musculoskeletal conditions were investigated in this study, provided they were associated with persistent pain lasting 12 weeks or more. During analysis, all inflammatory conditions were grouped together, including RA, AS, lupus, PsA and other less common inflammatory conditions such as palindromic rheumatism, scleroderma, DM, Sjögren’s and Crohn’s arthritis. Non-inflammatory conditions included OA, chronic lower back pain, FM, hypermobility and other less common non-inflammatory conditions such as, plantar fasciitis, post-traumatic chronic elbow pain, chronic post-traumatic knee pain, myalgic encephalomyelitis/chronic fatigue syndrome, frozen shoulder and tendinitis.

### Outcome measures

The study questionnaire included four main sections: demographic information, the Composite Autonomic Symptom Score 31 (COMPASS-31), the Brief Pain Inventory (BPI) and the Hospital Anxiety and Depression Scale (HADS) [10, 13, 14]. Participants were invited to complete the questionnaires either before or after their scheduled appointment and received a voucher as compensation for their time and participation.

The COMPASS-31 is a brief self-administered questionnaire designed to assess autonomic dysfunction symptoms [10]. It has been validated against the gold standard measure, the composite autonomic severity score (CASS), in different medical conditions and has proven its validity as an initial screening tool for dysautonomia [15, 16]. It comprises 31 items grouped into six domains, each reflecting a specific aspect of autonomic function. These domains include orthostatic intolerance, vasomotor, secretomotor, gastrointestinal, bladder and pupillomotor symptoms. Each item is scored based on its severity and frequency, resulting in a total score from 0 to 100. Higher scores indicate a greater degree of autonomic dysfunction [17]. A COMPASS-31 score of ≥17 out of a possible total of 100 indicated positive dysautonomia.

The Brief Pain Inventory-Short Form (BPI-SF) is a widely used tool for evaluating pain intensity and its impact on daily activities, requiring no >5 min to complete. Participants rate their pain intensity and its impact on daily activities (general activity, mood, walking, work, social interactions, sleep and enjoyment of life) on a scale from 0 to 10. These scores provide a comprehensive assessment of pain and assist healthcare providers in developing customized pain treatment strategies.

HADS is a validated instrument for assessing anxiety and depression symptoms in both the general population and patients with different medical conditions [14]. It contains seven questions and takes roughly 2–5 min to complete. It has two subscales: anxiety and depression. On each subscale, those scoring below 7 are classified as non-cases, while those scoring between 8 and 10 are classified as mild, 11–14 as moderate, and 15–21 as severe [18].

### Statistical approach

All participants were attending an outpatient musculoskeletal clinic and had received a formal diagnosis from hospital clinicians. Participants self-reported their primary musculoskeletal diagnosis during recruitment, which was categorized into two types—inflammatory or non-inflammatory CMP. Participants with more than one musculoskeletal diagnosis were classified based on the primary diagnosis they identified as most problematic and for which they were attending the clinic. Each participant was therefore assigned to a single condition and counted only once to avoid duplication. This enabled quantification of differences in prevalence estimates by condition type.

Participant’s demographic and clinical characteristics were summarized using category counts (%) and means (standard deviation), by type of musculoskeletal condition (inflammatory and non-inflammatory) and for all CMP conditions combined.

Linear regression models were used to quantify the difference in overall COMPASS-31 scores, and different COMPASS-31 domain scores, between types of musculoskeletal conditions. Logistic regression models were used to quantify the associations between pain severity, psychological factors (predictors) and dysautonomia (outcome). Relative risks (RRs) were derived from logistic regression using marginal standardization. Both unadjusted and adjusted models were performed, with adjusted models controlling for age and gender. Statistical significance was set at two-tailed *P* < 0.05. All analyses were carried out using Stata version 19 (StataCrop LLC, College Station, TX, USA).

### Sample size calculation

Since there was no prior research on dysautonomia prevalence in CMP patients, we used a conservative estimate of 33% for the expected prevalence based on previous studies of similar conditions [19, 20]. To provide a 95% CI for the prevalence of dysautonomia with a margin of error of ±5 percentage points, we required a final sample size of ∼340 individuals. Allowing for a 20% dropout rate, we aimed to recruit an initial sample of 408 individuals living with CMP.

### Ethical approval

Ethical approval of this study was obtained from Liverpool Central Research Ethics Committee (REC reference: 24/NW/0238) on 24 July 2024. The study was sponsored by the University of Leeds and conducted in accordance with Good Clinical Practice (GCP) guidelines and the UK Policy framework for Health and Social Care Research (2017). It is reported following the STROBE statement for cross-sectional studies (Supplementary Data S1).

## Results

**Figure 1:**
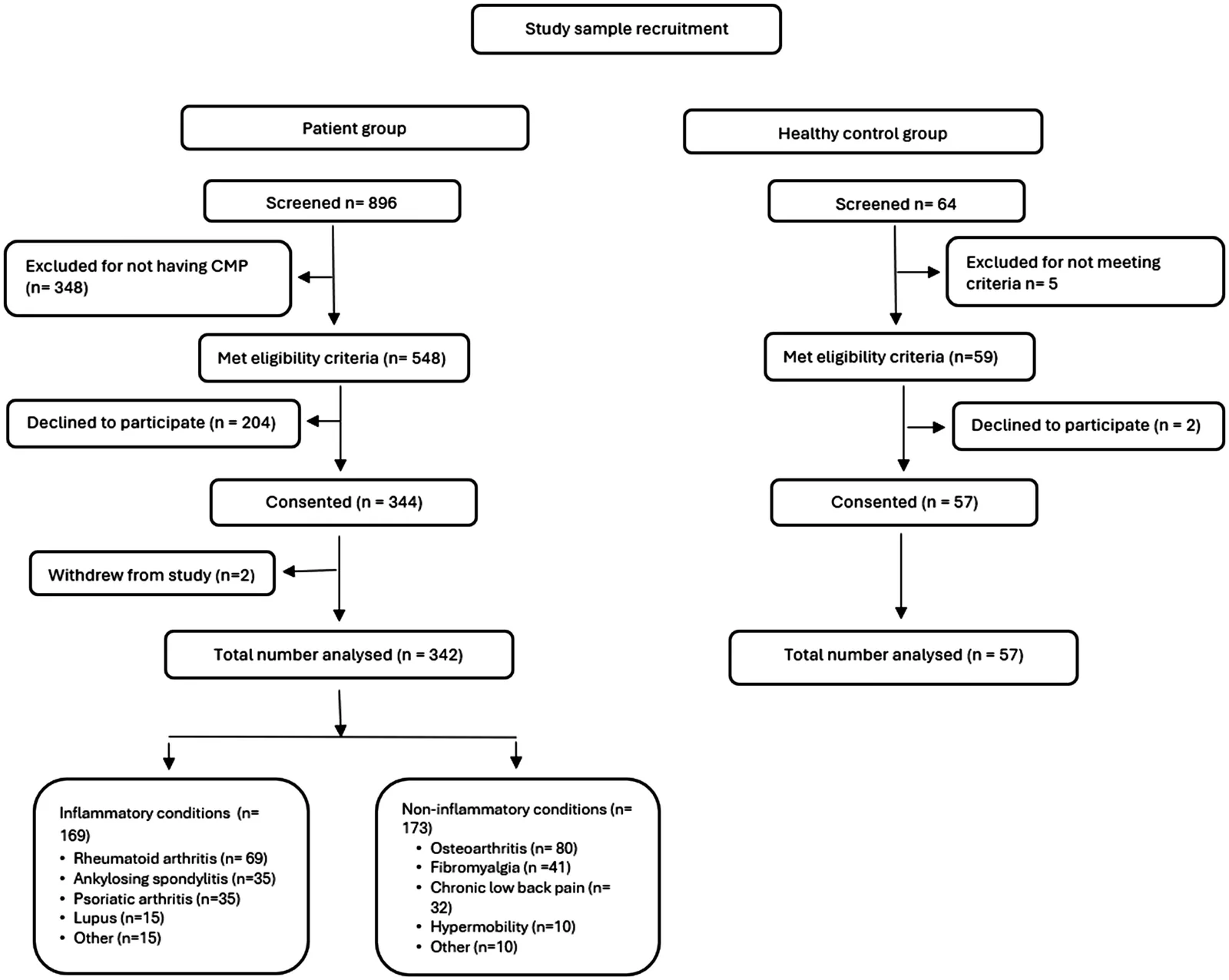
Study participant flow diagram

A total of 896 participants with painful musculoskeletal conditions were screened for eligibility; 548 met the inclusion criteria. Of these, 344 (63%) consented to participate, with 342 (62%) returning completed questionnaires. Based on their primary musculoskeletal diagnosis, 169 participants were identified as having an inflammatory condition, and 173 as non-inflammatory. Additionally, 57 healthy controls were recruited from 59 eligible (97%). The flow of participants through the recruitment process is presented in Fig. 1.

Most CMP participants were female (246; 72%), with a mean age of 48 years (S.D. 13) and predominantly White ethnicity (83%). Approximately one-fifth (22%) were employed in professional occupations. Similarly, healthy controls were predominantly female (77%) and White, with mean age of 44 years (S.D. 11). Detailed demographic characteristics are presented in Table 1.

**Table 1:** Demographic characteristics of participants returning questionnaires.

Demographic characteristics	Inflammatory (*n* = 169)	Non-inflammatory (*n* = 173)	All musculoskeletal conditions (*n* = 342)	Healthy control (*n* = 57)
Female gender, *n* (%)	120 (71%)	126 (73%)	246 (72%)	44 (77%)
Mean (S.D.) age (years)	48 (13.1)	49 (13.6)	48 (13.3)	44.3 (10.5)
Mean BMI (S.D.) (kg/m^2^)	28.7 (6.3)	30.4 (6.8)	29.5 (6.6)	25 (6)
Current smoker, *n* (%)	18 (11%)	24 (14%)	42 (12%)	1 (2%)
Ethnicity, *n* (%)				
White British, Irish, Irish Traveler or other White	140 (83%)	144 (83%)	284 (83%)	40 (70%)
Black African, Caribbean or other Black	10 (6%)	9 (5%)	19 (6%)	6 (11%)
Mixed	6 (4%)	3 (2%)	9 (3%)	1 (2%)
Asian/Asian British	12 (7%)	15 (9%)	27 (8%)	10 (18%)
Arab	1 (1%)	2 (1%)	3 (1%)	
Marital status, *n* (%)				
Single	53 (32%)	55 (32%)	108 (32%)	13 (23%)
Married or in domestic relationship	112 (66%)	111 (65%)	223 (65%)	42 (74%)
Prefer not to say	4 (2%)	6 (4%)	10 (3%)	2 (4%)
Job categories, *n* (%)				
Managers, directors and senior officials	33 (20%)	20 (12%)	53 (16%)	5 (9%)
Professional occupations	38 (24%)	33 (20%)	71 (22%)	29 (51%)
Associate professional and technical occupations	10 (6%)	10 (6%)	20 (6%)	7 (12%)
Administrative and secretarial occupations	14 (9%)	16 (10%)	30 (9%)	9 (16%)
Skilled trades occupations	9 (6%)	6 (4%)	15 (5%)	1 (2%)
Caring, leisure and other service occupations	14 (9%)	28 (17%)	42 (13%)	4 (7%)
Sales and customer service occupations	7 (4%)	7 (4%)	14 (4%)	
Process, plant and machine operations	2 (1%)	1 (0.64%)	3 (1%)	
Elementary occupations	6 (4%)	7 (4%)	13 (4%)	
Student	2 (1%)	7 (4%)	9 (3%)	2 (4%)
Retired	25 (15%)	29 (18%)	54 (17%)	

Two hundred and seventy-eight (81%) of participants had experienced CMP for more than 2 years. Pain significantly affected employment, with 165 (48%) reporting either reduced work hour or sick leave. The most common comorbidity was respiratory disease in 52 participants (15%). The most frequently reported symptoms were joint pain (*n* = 332, 97%), fatigue (*n* = 249,73%) and sleep disturbance (*n* = 245, 72%).

### Prevalence of dysautonomia in CMP

The median (IQR) COMPASS-31 score was 31.5 (IQR 16–46) (Table 2). Overall, 253 CMP participants (74%) met criteria for dysautonomia, with COMPASS-31 score ≥17. Dysautonomia prevalence was high in both inflammatory (76%, *n* = 128) and non-inflammatory (72%, *n* = 125) conditions. Females had significantly higher prevalence of dysautonomia (78%, *n* = 191) compared with males (65%, *n* = 62) (*P* = 0.013). Dysautonomia prevalence varied across different musculoskeletal conditions (Table 3) as well as across age groups, peaking at 84% in participants aged 40–49 years and ranging from 64% to 77% in other age groups.

**Table 2:** Clinical characteristics of participants returning questionnaires.

Clinical characteristics	Inflammatory (*n* = 169)	Non-inflammatory (*n* = 173)	All musculoskeletal conditions	Healthy control (*n* = 57)
Chronic pain duration, *n* (%)				
3–6 months	7 (4%)	4 (2%)	11 (3%)	
6 months to 1 year	11 (7%)	15 (9%)	26 (8%)	
1–2 years	10 (6%)	16 (10%)	26 (8%)	
+2 years	141 (83%)	137 (79%)	278 (81%)	
Reduced work hours or sick leave due to pain, *n* (%)	83 (49%)	82 (47%)	165 (48%)	
Comorbidities, *n* (%)				
Diabetes	18 (11%)	14 (8%)	32 (9%)	0 (0%)
Respiratory disease	25 (15%)	27 (16%)	52 (15%)	3 (5%)
Heart disease	4 (2%)	6 (3%)	10 (3%)	0 (0%)
Kidney disease	4 (2%)	8 (5%)	12 (4%)	0 (0%)
Liver disease	2 (1%)	1 (1%)	3 (1%)	0 (0%)
Neurological conditions	2 (1%)	2 (1%)	4 (1%)	0 (0%)
Key symptoms in the last 7 days *n* (%)				
Joint pain	163 (96%)	169 (98%)	332 (97%)	
Fatigue	120 (71%)	129 (75%)	249 (73%)	
Sleep disturbance	118 (69%)	127 (74%)	245 (72%)	
COMPASS-31				
Median (IQR) score	30 (17.6–44.8)	34 (15.9–48.4)	31.5 (16–46)	3 (0.9–9.3)
Dysautonomia, *n* (%)	128 (76%)	125 (72%)	253 (74%)	6 (11%)
Brief pain inventory				
Mean pain severity (0–10)	5.53 (2)	5.90 (2)	6 (2)	0.6 (1.6)
Mean pain interference (0–10)	5.57 (3)	6.19 (3)	6 (3)	0.4 (1.2)
HADS				
Median (IQR) anxiety score	10 (6–13)	11 (6–14)	10 (6–14)	3 (1-6)
Anxiety cases, *n* %	78 (46%)	92 (53%)	170 (50%)	3 (5%)
Median (IQR) depression score	8 (5–12)	9 (5–12)	9 (5–12)	1 (0–3)
Depression cases, *n* %	62 (37%)	69 (40%)	131 (38%)	2 (4%)

**Table 3:** Dysautonomia prevalence by study group.

Study group	Sample size	No dysautonomia, *n* (%)	Dysautonomia cases, *n* (%)
Inflammatory conditions			
RA	69	22 (31.9%)	47 (68.1%)
AS	35	11 (31.4%)	24 (68.6%)
PsA	35	5 (14.3%)	30 (85.7%)
Lupus	15	0 (0%)	15 (100%)
Other inflammatory^a^	15	3 (20%)	12 (80%)
Inflammatory subtotal	169	41 (24.3%)	128 (75.7%)
Non-inflammatory conditions			
OA	80	29 (36.3%)	51 (63.8%)
Chronic lower back pain	32	9 (28.1%)	23 (71.9%)
FM	41	2 (4.9%)	39 (95.1%)
Hypermobility	10	0 (0%)	10 (100%)
Other non-inflammatory^a^	10	8 (80%)	2 (20%)
Non-inflammatory subtotal	173	48 (27.7%)	125 (72.3%)
Healthy controls	57	51 (89.5%)	6 (10.5%)

a Other non-inflammatory include frozen shoulder, post-traumatic chronic elbow pain, post-traumatic chronic knee pain, myalgic encephalomyelitis/chronic fatigue syndrome, tendinitis and plantar fasciitis. Other inflammatory include: palindromic rheumatism, scleroderma, DM, Sjögren’s and Crohn’s arthritis.

Table 4 presents all the COMPASS-31 domain scores stratified by type of musculoskeletal pain. Participants with non-inflammatory conditions reported greater severity of dysautonomia across all COMPASS-31 domains (mean = 34.6, S.D. = 21.3) compared with those with inflammatory conditions (mean = 30.7, S.D. = 17.9); however, this difference of 3.9 points (95% CI −0.3 to 8, *P* = 0.066) was not statistically significant after adjusting for age and gender. This difference was primarily driven by significantly higher scores in the gastrointestinal and bladder domains (*P* = 0.042 and *P* = 0.005, respectively).

**Table 4:** COMPASS-31 domain scores by study group.

Domain	Max weighted score	Healthy controls	Inflammatory	Non-inflammatory	All musculoskeletal	Unadjusted difference	*P*	Adjusted difference^a^	*P*
		Mean (S.D.)	Mean (S.D.)	Mean (S.D.)	Mean (S.D.)	(95% CI)		(95% CI)	
Orthostatic intolerance	40	2.8 (5.7)	14.6 (11.2)	15.9 (12.4)	15.2 (11.9)	1.3 (−1.2 to 3.8)	0.309	1.3 (−1.2, 3.8)	0.307
Vasomotor	5	0.1 (0.5)	1.3 (1.7)	1.4 (1.8)	1.4 (1.7)	0.1 (−0.2 to 0.5)	0.506	0.1 (−0.2, 0.5)	0.491
Secretomotor	15	0.5 (1.3)	4.4 (4.0)	5 (3.8)	4.7 (3.9)	0.7 (−0.2 to 1.5)	0.117	0.6 (−0.2, 1.4)	0.161
Gastrointestinal	25	1.8 (2.6)	7.5 (4.9)	8.6 (5.4)	8.1 (5.2)	1.1 (0.0–2.2)	0.050	1.1 (0.0, 2.2)	0.042
Bladder	10	0.2 (0.5)	1.2 (1.7)	1.8 (2.2)	1.5 (2.0)	0.6 (0.2–1.0)	0.004	0.6 (0.2, 1.0)	0.005
Pupillomotor	5	0.6 (0.7)	1.7 (1.2)	1.9 (1.4)	1.8 (1.3)	0.2 (−0.1 to 0.4)	0.263	0.1 (−0.1, 0.4)	0.290
Total COMPASS-31	100	6 (7.1)	30.7 (17.9)	34.6 (21.3)	32.7 (19.8)	4.0 (−0.2 to 8.2)	0.063	3.9 (−0.3, 8.0)	0.066

a Adjusted for age and gender. Differences represent non-inflammatory minus inflammatory group scores.

Overall, the pattern of dysautonomia symptoms was similar between the condition groups, though non-inflammatory conditions showed numerically greater symptom severity. The COMPASS-31 domain scores broken down further by specific condition type are shown in Table 5.

**Table 5:** COMPASS-31 domains scores by specific musculoskeletal condition.

Condition	Orthostatic intolerance (max 40)	Vasomotor (max 5)	Secretomotor (max 15)	Gastrointestinal (max 25)	Bladder (max 10)	Pupillomotor (max 5)	Total COMPASS-31 (max 100)
Inflammatory							
RA (*n* = 69)	13.4 (11.2)	0.8 (1.4)	3.8 (4)	6 (4.4)	0.7 (1.2)	1.5 (1.1)	26.3 (16.7)
AS (*n* = 35)	12.9 (11.8)	0.9 (1.5)	4 (3.5)	7.5 (4.1)	1.1 (1.3)	1.6 (1.1)	28.1 (17.4)
PsA (*n* = 35)	15.7 (11.3)	1.5 (1.7)	4.9 (4.4)	8.2 (5.5)	1.6 (2.2)	1.9 (1.3)	33.7 (17.9)
Lupus (*n* = 15)	23.2 (7.3)	2.8 (1.7)	7.3 (3.1)	9.1 (5.8)	3 (2.7)	2.7 (1.4)	48.1 (17)
Other inflammatory (*n* = 15)	12.5 (9.8)	2.7 (1.4)	3.4 (3.5)	11.3 (4.4)	0.7 (0.9)	1.7 (1.3)	32.3 (15.5)
Non-inflammatory							
OA (*n* = 80)	13.4 (12)	1.3 (1.7)	4.8 (3.6)	7.9 (5.3)	1.6 (2.1)	1.7 (1.3)	30.6 (20.1)
FM (*n* = 41)	24.2 (10.3)	2.3 (2)	6.4 (4)	12.1 (4.4)	2.7 (2.4)	2.5 (1.4)	50.3 (18.2)
Chronic low back pain (*n* = 32)	13 (11.1)	0.8 (1.3)	4.1 (3.3)	6.6 (5.2)	1.1 (1.4)	1.6 (1.2)	27.2 (15.8)
Hypermobility (*n* = 10)	22.8 (6.3)	1.9 (2.1)	5.8 (3.5)	9.6 (4.1)	1.9 (1.9)	2.3 (1.2)	44.2 (13.7)
Other non- inflammatory (*n* = 10)	4 (12.6)	1.1 (1.5)	3.2 (4.1)	6.2 (6.6)	1.6 (2.8)	1.2 (1.6)	17.2 (26.2)

Values are mean (S.D.) of weighted domain scores. Higher scores indicate greater autonomic symptom burden.

### Exploratory analyses of comorbid conditions

Eleven participants reported gastrointestinal conditions in the free-text field (inflammatory bowel disease, Crohn’s disease, colitis, irritable bowel syndrome or colic disease). Compared with the remaining participants, these participants showed similar total COMPASS-31 scores (median 29 compared with 31.6, *P* = 0.74) and a similar dysautonomia prevalence (72.7% compared with 74%). Gastrointestinal domain scores were modestly higher in this group (median 10.7 compared with 8), but not statistically significant (*P* = 0.38).

FM was reported by 59 participants, of whom 41 had it as their index diagnosis and 18 reported it as a comorbid condition. Participants with comorbid FM had higher total COMPASS-31 scores than the 283 participants without FM (median 43.7 compared with 28.7, *P* = 0.006) and a higher prevalence of dysautonomia (88.9% compared with 70%). These 18 participants were evenly distributed between inflammatory (*n* = 9) and non-inflammatory conditions (*n* = 9). Excluding them only changed the overall prevalence of dysautonomia marginally from 74% to 73.2%.

### Dysautonomia prevalence in healthy controls

Healthy controls demonstrated low COMPASS-31 scores across all domains compared with cases (Table 4), with a median (IQR) of 3 (0.9–9.3), and six controls (11%) meeting dysautonomia criteria with COMPASS-31 score ≥17. CMP cases were substantially more likely to meet dysautonomia criteria than healthy controls (unadjusted RR = 7.0, 95% CI 1.7–12.4, *P* = 0.010; adjusted RR = 7.4, 95% CI 1.8–13.1, *P* = 0.010). Similarly, HADS scores were low, and pain levels were minimal in the controls group (Table 2).

### Pain severity, anxiety and depression

HADS scores indicated anxiety in 170 participants (50%) and depression in 131 (38%) (Table 2). The median HADS anxiety score was 10 (6–14) and HADS depression score was 9 (5–12).

Table 6 shows logistic regression results for pain severity and psychological factors as predictors of dysautonomia in CMP cases. Adjusted models controlled for age and gender. Higher pain severity was significantly associated with increased risk of dysautonomia; every 1-point increase in pain severity, the risk of dysautonomia increased by 27% (RR 1.27, 95% CI 1.08–1.45, *P* < 0.001) after adjusting for age and gender. Similarly, psychological factors demonstrated significant associations: each 1-point increase in depression score was associated with a 12% increase in the risk of dysautonomia (RR 1.12, 95% CI 1.06–1.19, *P* < 0.001), while each 1-point increase in anxiety score corresponded to a 15% increase in the risk of dysautonomia (RR 1.15, 95% CI 1.08–1.22, *P* < 0.001), after controlling for the same confounders.

**Table 6:** Predictors of dysautonomia in CMP (*N* = 342).

Variable	Unadjusted RR (95% CI)	*P*-value	Adjusted^a^ RR (95% CI)	*P*-value
Pain severity (per point)	1.28 (1.09–1.46)	<0.001	1.27 (1.08–1.45)	<0.001
Depression score (per point)	1.13 (1.06–1.20)	<0.001	1.12 (1.06–1.19)	<0.001
Anxiety score (per point)	1.16 (1.08–1.23)	<0.001	1.15 (1.08–1.22)	<0.001

a Adjusted for age and gender. Relative risks derived from logistic regression using the margins method.

## Discussion

This study addressed the research gap of investigating the prevalence of dysautonomia across different musculoskeletal conditions associated with chronic pain. Nearly three-quarters of CMP participants were classified as having dysautonomia based on COMPASS-31 criteria, regardless of the nature of their underlying condition, either inflammatory or non-inflammatory. However, participants with non-inflammatory conditions reported greater severity of dysautonomia compared with those with inflammatory conditions, although this difference did not reach statistical significance. Scores in the GI and bladder domains were significantly higher in non-inflammatory conditions, but the absolute differences were small. The bladder domain difference of 0.6 points, on a domain with a maximum weighted score of 10, may not be noticeable by the patient.

Females had a significantly higher prevalence of dysautonomia compared with males, consistent with the literature and clinical experience [21, 22]. Participants with higher pain severity, anxiety and depression scores were more likely to have dysautonomia. These associations remained even after adjusting for age and gender, suggesting that these factors are independent predictors of dysautonomia in this population.

Our research confirms earlier work by Puri and Lee [17], in a sample of 25 people living with FM, showing that the condition is coincident with autonomic dysfunction captured using COMPASS 31. Our work builds on this further by validating these findings in a larger sample and extending the results to other musculoskeletal conditions, both non-inflammatory and inflammatory. The association between dysautonomia and CMP severity observed in this study is consistent with previous investigations [11]. The high prevalence of dysautonomia in our sample of patients is also comparable to a recent study in females with FM [12]. However, previous studies of dysautonomia symptoms in RA patients reported 47% of positive cases [23], compared with 68% in our RA subgroup. This difference may reflect methodological differences, as Louthrenoo *et al.* [23] used cardiovascular reflex tests in a smaller sample (*n* = 34) compared with COMPASS 31 in our study. Similar discrepancies were observed with lupus, where an earlier study reported 37% prevalence going by symptoms, and 18% prevalence using objective testing methods [24], compared with the 100% in our study Table 3.

Our findings should be interpreted in light of several methodological limitations. First of all, in common with previous studies using a cross-sectional design, and despite potential mechanistic evidence [25], we are unable to infer direct causality. Additionally, our assessment of dysautonomia relied on self-reported symptoms, rather than objective testing. Subjective and objective measures of dysautonomia do not always agree and a mismatch between symptom-based and physiological assessment has been reported [26]. Our prevalence estimates should be interpreted as reflecting autonomic symptom burden rather than confirmed autonomic dysfunction, and future study should seek to confirm these findings using objective autonomic testing.

Secondly, whilst the diagnostic threshold for dysautonomia we applied (COMPASS-31 ≥ 17) is well-established [27], and COMPASS-31 has been used in chronic musculoskeletal conditions research [28, 29], this specific cut-off has not been validated in CMP population yet.

Although we recorded many comorbid conditions at recruitment, gastrointestinal conditions such as inflammatory bowel disease and Crohn’s disease were not included as a direct item in our comorbidity checklist and could not be systematically assessed. Reliance on spontaneous reporting (in the free-text option) means that gastrointestinal comorbidity may be underestimated and its influence cannot be fully excluded. Post-infection conditions were similarly not recorded as a direct item. Two participants reported myalgic encephalomyelitis/chronic fatigue syndrome as their index musculoskeletal condition and one participant reported long COVID in the free-text field. These conditions were not systematically screened for, and their presence therefore cannot be excluded and may have contributed to the autonomic symptom burden observed in this cohort. Gastrointestinal conditions affect the gut-immune-brain axis, and post- infection syndromes are an established cause of dysautonomia; future studies should capture both gastrointestinal and post-infection conditions systematically.

Participants could report all their musculoskeletal conditions, but each was assigned to a single index diagnosis. Although comorbid FM was associated with a greater autonomic symptom burden, these participants were evenly distributed between inflammatory and non-inflammatory conditions and excluding them altered the overall prevalence of dysautonomia only marginally. These exploratory comparisons were based on a small number of participants and warrants further investigation in larger samples.

A further limitation is the absence of systemic medication data. Participants were asked only about the medication they received for pain, and this was not intended to capture full medication history. As a result, the use of DMARDs and biologic therapies could not be reliably determined. Given that such treatments modulate inflammatory mediators and could plausibly affect autonomic symptoms, their potential influence on COMPASS-31 scores remains unclear and requires systematic investigation in future work.

Our study sample was recruited from a single site with a predominantly white ethnicity. This may limit the generalizability of our findings to the broader CMP population globally. Furthermore, whilst we had an overall large sample size, the study was powered to estimate the prevalence of dysautonomia rather than to detect differences between condition subgroups. Therefore, secondary comparisons between inflammatory and non-inflammatory conditions such as COMPASS-31 scores should be interpreted as exploratory and may be underpowered. Also, the small sample sizes for certain sub-types limit our ability to compare individual conditions, such as lupus and hypermobility. A higher proportion of patients than healthy controls declined to participate (37% compared with 3%). This partly reflects that a large number of patients were approached during recruitment compared with a relatively small number of healthy controls. It also likely reflects the greater symptom burden and fatigue experienced around their clinic appointments.

Despite these limitations, our research has several key strengths, including addressing a significant gap in the literature by quantifying dysautonomia prevalence in CMP compared with healthy controls and analysing dysautonomia across both inflammatory and non-inflammatory musculoskeletal conditions. In addition, we have used comprehensive, validated and time-efficient self-reported tools to reflect real-world symptoms burden for dysautonomia, pain and psychological factors. Additionally, the large sample size provided good overall statistical power, strengthened the validity of the findings and facilitated statistical comparability between inflammatory and non-inflammatory chronic pain conditions.

Our study provides evidence against the belief that dysautonomia is uncommon and seen predominantly in non-inflammatory conditions. The high prevalence of symptoms consistent with dysautonomia suggest that routine screening for dysautonomia should be carried out in all CMP patients. Screening can be conducted using subjective measures (such as COMPASS 31) followed by objective tests (such as a 10 min Lean Test or Tilt Table test) [30].

Our findings validate the symptoms experienced by individuals with CMP and provide further insights into its pathophysiology. Our study will help understand persistent pain and fatigue symptoms in many patients where the primary pathology has been treated and well-controlled. There is a need for further research into the link between dysautonomia and pain in these patients and the effect of dysautonomia-based treatment programmes for such persistent symptoms. There is some existing literature on the use of non-pharmacological and pharmacological treatments for dysautonomia [31].

Future work should also focus on the physiological basis of dysautonomia in clarifying the direction of causality between dysautonomia and CMP. The interaction between ANS, the hypothalamic–pituitary–adrenal (HPA) axis and immune system is well recognized in the literature [32]. Longitudinal studies in CMP will help understand the temporal relationship better and the development of dysautonomia. Such an understanding will help develop potential treatments directed towards dysautonomia that could improve overall symptom burden and well-being in this population.

## Supplementary Material

keag511_Supplementary_Data

## Data Availability

Data are available upon reasonable request to the corresponding author.
